# Suicide and depression in former contact sports participants: population-based cohort study, systematic review, and meta-analysis

**DOI:** 10.1016/j.eclinm.2023.102026

**Published:** 2023-06-08

**Authors:** G. David Batty, Philipp Frank, Urho M. Kujala, Seppo J. Sarna, Jaakko Kaprio

**Affiliations:** a Department of Epidemiology and Public Health, University College London, London, UK; b Faculty of Sport and Health Sciences, University of Jyväskylä, Jyväskylä, Finland; c Department of Public Health, University of Helsinki, Helsinki, Finland; d Institute for Molecular Medicine FIMM, University of Helsinki, Helsinki, Finland

**Keywords:** Suicide, Depression, Contact sports, Head impact, Cohort study, Systematic review, Meta-analysis

## Abstract

**Background:**

Former participants in sports characterised by low intensity repetitive head impact appear to have elevated rates of later dementia, but links with other psychological health outcomes such as depression and suicide are uncertain. We quantified the occurrence of these endpoints in former contact sports athletes against general population controls using new data from a cohort study and a meta-analysis.

**Methods:**

The cohort study comprised 2004 retired male athletes, who had competed internationally as amateurs for Finland across a range of sports, and 1385 general population controls. All study members were linked to mortality and hospitalisation registries. In the PROSPERO-registered systematic review (CRD42022352780), we searched PubMed and Embase to October 31 2022 for cohort studies that reported standard estimates of association and precision. Study-specific estimates were aggregated in a random-effect meta-analysis. The Newcastle-Ottawa Scale was used to appraise the quality of each study.

**Findings:**

In survival analyses of the Finnish cohort data, former boxers (depression: hazard ratio 1.43 [95% CI 0.73, 2.78]; suicide: 1.75 [0.64, 4.38]), Olympic-style wrestlers (depression: 0.94 [0.44, 2.00]; suicide: 1.60 [0.64, 3.99]), and soccer players (depression: 0.62 [0.26, 1.48]; suicide: 0.50 [0.11, 2.16]) did not have statistically higher rates of major depressive disorder or suicide at follow-up relative to controls. In the systematic review, 7 cohort studies met inclusion criteria. After aggregating results with the Finnish cohort, retired soccer players appeared to have a lower risk of depression (summary risk ratio: 0.71 [0.54, 0.93]) relative to general population controls, while the rate of suicide was statistically the same across groups (0.70 [0.40, 1.23]). Past participation in American football seemed to be associated with some protection against suicide (0.58 [0.43, 0.80]) but there were insufficient studies of depression in this sport to facilitate aggregation. The aggregation of results from the soccer and American football studies showed directionally consistent relationships and there was no indication of inter-study heterogeneity (I^2^ = 0%).

**Interpretation:**

Based on a small cluster of studies exclusively comprising men, retired soccer players had a lower rate of later depression and former American football players had a lower risk of suicide relative to comparator groups. Whether these findings are generalisable to women requires testing.

**Funding:**

The preparation of this manuscript was unfunded.

**Research in context**

**Evidence before this study**

Former participants in sports characterised by low intensity repetitive head impact appear to have elevated rates of later dementia, but links with other psychological health outcomes such as depression and suicide are uncertain. Searching electronic databases using terms for specific contact sports (e.g., ‘soccer’, ‘wrestling’), depression, and suicide revealed a small series of studies of former soccer players and American footballers, and none on erstwhile athletes from combat sports (e.g., wrestling or boxing). There was also no meta-analytical aggregation of the evidence.

**Added value of this study**

Taking new results from individual-participant analysis of a cohort of retired amateur athletes together with the existing literature, there was no suggestion that retired soccer and American football players had poorer mental health or elevated suicide risk relative to the general population. Rather, in the few qualifying studies, we found that erstwhile soccer players appeared to have a lower risk of depression relative to general population controls, while former American football players appeared to experience some protection against suicide.

**Implications of all the available evidence**

The potential for American football (suicide) and soccer (depression) to seemingly impact positively on psychological health outcomes in the present aggregation of a modest evidence base requires further testing, most obviously in women.

## Introduction

A series of large-scale cohort studies reveal a higher rate of suicide and depression in individuals with a history of traumatic brain injury severe enough to require hospitalisation relative to unaffected population controls.^1^^,^^2^ In the more comprehensive investigations, these effects seem to be independent of measured confounding factors, including comorbidities.^3^ These observations raise the possibility that a history of involvement in sports characterised by repetitive low-level head impact, such as boxing, soccer, and American football, might be linked to the development of depression and suicide, as has recently been advanced for other indicators of psychological health such as dementia and Alzheimer’s disease.^4^

Much of the evidence for contact sports having an impact on depression and suicide stems from case reports of select athlete samples where a post-mortem diagnosis of chronic traumatic encephalopathy, formerly termed dementia pugilistica, is reportedly accompanied by depression, suicide, and/or aggressive behaviours.^5^^,^^6^ These results are at best hypothesis-generating, and any potential link between past participation in contact sports, depression, and suicide requires testing in cohort studies in which the long-term health experience of retired athletes is compared with unaffected population controls.^7^ Such studies are rare however, and seem to reveal discordant findings such that a lower incidence of depression in retired soccer players^8^ and suicide in former American football professionals^9^ has been reported relative to unexposed individuals, while in other studies, no such group differences were evident.^10^^,^^11^ It is plausible that the contrasting profile of head impacts across different contact sports may account for the apparently different pattern of disease risk but such cross-sport comparisons are currently lacking.^12^

We address these uncertainties in two ways. We first report new results from a cohort of retired amateur athletes representing an array of elite-level sporting backgrounds, and then integrate these findings into a meta-analysis based on a systematic review of the available literature. To the best of our knowledge, there is no existing meta-analysis of depression in former contact sports athletes, and in a recent aggregation of suicide results from studies of retired soccer and American football players, there was a suggestion that these occupations conferred some protection.^13^ No sport-specific estimates were provided, however. That global participation in soccer—estimated at more than a quarter of a billion by its governing body^14^—is seemingly the highest of any sport, and programmes of American football are long-established in some educational institutions,^15^ means that a link between a background in these activities and depression or suicide may have public health relevance.

## Methods

### Cohort of Finnish former elite athletes and population controls

This cohort study was initiated in 1978 to examine the relationship between participation in sports and long-term health.^16^, ^17^, ^18^, ^19^ In brief, former athletes were selected based on the following criteria: male; represented Finland 1920–1965 on at least one occasion in the Olympic games, World or European championships, or intercountry competitions; and competed in track and field athletics, cross-country skiing, soccer, ice hockey, basketball, boxing, wrestling, weight-lifting, or shooting. Full name, and place and date of birth were extracted from sports yearbooks and registers of sports associations, and, if necessary, enquiries were made to relatives, friends, sports journals, and Finnish embassies abroad. This process resulted in a group 2613 men and represented the athlete cohort.

A population-based comparison group was identified using a database generated from the medical examination for induction into military or civic service which was, and remains, compulsory for all men in Finland. For a referent to be selected, he needed to be aged 20 years, apparently healthy (classified as ‘A1' in the database), and from the same area of residence as the comparator athlete. After first locating the athlete in the population register, the most proximate control matching these inclusion criteria was then selected. This resulted in general population comparison group of 1712 men. Men in the athlete cohorts were not included in the control group.

Data collection was approved by the ethics committee of the Hospital Districts of Helsinki and Uusimaa, and all participants consented. The reporting of this cohort study conforms to the Strengthening the Reporting of Observational Studies in Epidemiology (STROBE) Statement of guidelines for the presentation of observational studies.^20^

#### Derivation of exposed and unexposed groups

In the absence of data on frequency of all head impacts combined, we used concussion occurrence as a proxy (summarised in Supplemental Table S1).^21^, ^22^, ^23^ Contact sports were grouped as soccer, boxing, wrestling, ice hockey, and basketball, and non-contact as track and field, cross-country skiing, and weight-lifting. Individual contact sports were then disaggregated as the numbers of depression and suicide cases at follow-up allowed in our analyses. Thus, separate analyses were possible for former soccer players, boxers, and wrestlers (non-professional or freestyle/Greco-Roman), while retired athletes from the sports of ice hockey and basketball players were combined into a ‘other’ contact sports category.

#### Assessment of covariates

Data on covariates were extracted from two sources. The presence of diabetes, hypertension, and coronary heart disease was derived from linkage of study members to a national drug treatment register. Additionally, in 1985, surviving study members and population controls (N = 2851; 66% of the original cohort) were mailed a self-completion questionnaire (N = 1917; response 67%) with enquiries regarding health behaviours (smoking, alcohol intake), physical stature, and weight. Questionnaire data in combination with those extracted from the Finnish Central Population Registry were used to generate a variable for longest held job, our indicator of socioeconomic status.^24^

#### Ascertainment of depression, depression ‘caseness’, and suicide

Health surveillance of study members began upon initiation of nationwide health registries in Finland in 1970 when the average age of the athlete group was 45.4 years (controls 44.3 years). Study members were linked to death (suicide) and hospitalisation (depression and suicide) records. Major depressive disorder was coded according to the International Classification of Disease (ICD) version eight (29600, 29620, 30040, 30041), nine (2961, 2968A, 3004A), or ten (F32–F34). The ICD codes used to denote suicide were E950–E959 (version eight and nine), or X60–X84 (version ten).

As part of another mailed questionnaire survey in 1995, surviving study members completed the Brief Symptom Inventory,^25^ a 53 item scale of psychological distress. For each of the 6 items that comprise the depression subscale, respondents used a 5 point continuum (0–4) to indicate the extent to which they had been concerned by suicidal ideation, loneliness, or a lack of interest in usual activities in the prior week (total 0–24).^25^ We used a score of ≥11 to denote depression ‘caseness’. Based on analyses of data from the Finnish cohort, this threshold was strongly associated with subsequent risk of hospitalisation for major depressive disorder (age-adjusted odds ratio: 6.41 [95% CI 3.82, 10.75]) and suicide (age-adjusted hazard ratio: 7.58 [95% CI 2.33, 24.69]) in the expected direction, suggesting some predictive validity.

### Systematic review and meta-analysis

#### Search strategy and study selection

This PROSPERO-registered (CRD42022352780) systematic review and meta-analysis is presented in accordance with the guidelines for Preferred Reporting Items for Systematic reviews and Meta-Analyses (PRISMA).^26^ We identified relevant literature by searching PubMed (Medline) and Embase databases between their inception and October 31, 2022. We used combinations of free text and controlled terms in 2 categories (Supplemental Box S1): the exposure (e.g., specific sports such as boxing, soccer, martial arts, and rugby), and the outcome (e.g., depression and suicide). We also scrutinised the reference sections of retrieved articles for additional publications.

We included a published paper if it fulfilled the following criteria: utilised a cohort study design; the identification of former participants in contact sports was records-based (e.g., pension or union registers, association or school/college yearbooks) rather than being self-declared; a comparison of depression and/or suicide occurrence was made between a group of former contact sports athletes and unexposed (general population) or lesser-exposed (former athletes from non-contact sports) controls; standard estimates of association (e.g., relative risk, odds ratios, hazard ratios) and precision (e.g., confidence interval, standard error) were reported or could be calculated based on the occurrence of depression and/or suicide using these data; published in a peer-reviewed journal; and published in English. Two authors (GDB and PF) independently screened the identified records first by title, then abstract, and, if necessary, the full paper. There were no discrepancies of note.

We classified sports participation as professional (salaried) or amateur (non-salaried). We reasoned that individuals who were professional—that is, for whom sport was their primary occupation—would be exposed to a greater number of head impacts in training and probably competition relative to amateurs. Elite participation does not necessarily imply a professional (salaried) individual; rather, it is dependent on the epoch of participation. For example, for those athletes who achieved the pinnacle of their sport by representing their country in the Olympic games, this would in fact have been on an amateur basis prior to 1986 when professional athletes were admitted.

#### Extraction of results and assessment of study quality

Where available, a range of characteristics were extracted from each publication, including the name of the lead author, publication year, country of sample population, number of exposed and unexposed participants, number of events, and effects estimates from both minimally- and multivariable-adjusted analyses. Study authors were contacted when clarification was required.

We used the Newcastle-Ottawa Scale to appraise the quality of each study (Supplemental Table S2).^27^ Comprising eight domains, including the comprehensiveness of exposure and outcome ascertainment, and adequacy of the period of health surveillance, a higher score denoted better quality (maximum 9). Studies with a score of ≥7 were regarded as high grade.

### Statistical analyses

In individual-participant analyses of the Finnish cohort study, after exclusion of study members owing to record-linkage failure and death prior to the beginning of follow-up, the main analytical sample comprised 3389 men (2004 former athletes, 1385 population controls). Event surveillance was from 1st January 1970 until the occurrence of a depression or suicide event or the end of the surveillance period (December 31, 2015)—whichever came first. Having ascertained that the proportional hazards assumption had not been violated, we used Cox regression to compute hazard ratios with accompanying 95% confidence intervals to summarise the relationship of a background in contact sports with later risk of depression and suicide.^28^ Age was used as the time covariate in the most basic model, with other covariates subsequently added, including indicators of socioeconomic status, co-morbidity, and health behaviours, all of which have been linked to depression and/or suicide risk.^29^ We used linear regression to compute beta coefficients with accompanying 95% confidence intervals for the continuously scored depression index from the Brief Symptom Inventory.

For the meta-analysis, we pooled the results from analyses of the Finnish cohort alongside published study-specific estimates using a random effects meta-analysis,^30^ an approach which incorporates the heterogeneity of effects in the computation of their aggregation. An I^2^ statistic was computed to summarise the heterogeneity in estimates across studies. Individual-participant analyses were performed using Stata 15 (StataCorp, College Station, TX), and the meta-analysis was conducted using R.

### Role of funding

The preparation of this manuscript was unfunded.

## Results

### Finnish cohort study

In analyses of the Finnish cohort data, up to 45 years of health event surveillance in an analytical sample of 3389 men gave rise to 131 hospitalisations for major depressive disorder, and 61 suicides (20 attempts, 41 deaths). A subgroup of 1419 men responded to the Brief Symptom Inventory. Taken together, there was no clear evidence of an association between a history of contact sports participation and later risk of major depressive disorder (Table 1), such that the confidence intervals for all point estimates included unity. Based on the subgroup of participants who responded to questionnaire enquiries about depression symptoms, there was a lower depression score amongst one-time wrestlers (age- and socioeconomic status -adjusted beta coefficient [95% confidence interval]: −1.04 [−1.92, −0.17]). A lower depression symptom score was also apparent, however, in athletes who formerly engaged in non-contact sports (−0.69 [−1.22, −0.15]). Analyses in which we computed odds ratios based on depression caseness for the purposes of inclusion in the meta-analysis produced a similar pattern of results (Supplemental Table S3).

**Table 1:** Association of participation in contact sports with hospitalisation for major depressive disorder and self-reported depression score: Finnish cohort study.

	Major depression disorder (hospitalisation) (hazard ratios [95% confidence intervals])			Depression score (Brief Symptom Inventory) (beta coefficients [95% confidence intervals])	
	Number of events/number at risk	Age-adjustment	Age- and SES-adjustment	Age-adjustment (N = 1419)	Age- and SES-adjustment (N = 1395)
Boxing	11/230	1.43 (0.74, 2.75)	1.43 (0.73, 2.78)	0.36 (−0.59, 1.31)	0.15 (−0.81, 1.11)
Wrestling	8/247	0.94 (0.45, 1.99)	0.94 (0.44, 2.00)	−0.98 (−1.85, −0.10)	−1.04 (−1.92, −0.17)
Soccer	6/248	0.64 (0.27, 1.49)	0.62 (0.26, 1.48)	−0.81 (−1.63, 0.01)	−0.59 (−1.43, 0.25)
Other contact sports	8/236	0.77 (0.36, 1.62)	0.75 (0.34, 1.63)	−1.08 (−1.83, −0.32)	−0.67 (−1.46, 0.13)
Non-contact sports	49/1043	1.24 (0.83, 1.85)	1.22 (0.80, 1.87)	−0.93 (−1.44, −0.42)	−0.69 (−1.22, −0.15)
General population (controls)	49/1385	1.00 (ref)	1.00 (ref)	0.00 (ref)	0.00 (ref)

In the analyses of suicide events in the Finnish cohort study (Table 2), again, there was no clear suggestion of an association with prior participation in any of the contact sports depicted. Confidence intervals were also wide on occasion indicating low statistical power owing to a very small number of events for selected sports.

**Table 2:** Hazard ratios (95% confidence intervals) for the association of participation in contact sports with suicide: Finnish cohort study.

	Number of events/number at risk	Age-adjustment	Age- and SES-adjustment
Boxing	6/230	1.57 (0.65, 3.84)	1.75 (0.64, 4.38)
Wrestling	6/247	1.44 (0.59, 3.53)	1.60 (0.64, 3.99)
Soccer	2/248	0.43 (0.10, 1.83)	0.50 (0.11, 2.16)
Other contact sports	3/236	0.54 (0.16, 1.79)	0.61 (0.17, 2.13)
Non-contact sports	19/1044	1.00 (0.55, 1.82)	1.14 (0.60, 2.20)
General population (controls)	25/1386	1.00 (ref)	1.00 (ref)

Lastly, we carried out some sensitivity analyses. Where they occured, the somewhat lower rates of depression and suicide in former contact sports athletes could be ascribed to their more favourable risk factor profile. That is, relative to the general population, post-retirement, athletes tended to have a lower prevalence of smoking, heavy alcohol intake, and socioeconomic deprivation, and it could be these factors rather than their status as former contact sports participants that lowers their risk of suicide and depression. The questionnaire mailed in 1985 captured these covariates and in analyses in which we collapsed all contact sports participants into a single group to preserve statistical power (68 cases of major depressive disorder in follow-up of 1897 men, and 20 cases of suicide in 1913 men from 1985), our conclusions were unchanged.

### Systematic review and meta-analysis

**Fig. 1:**
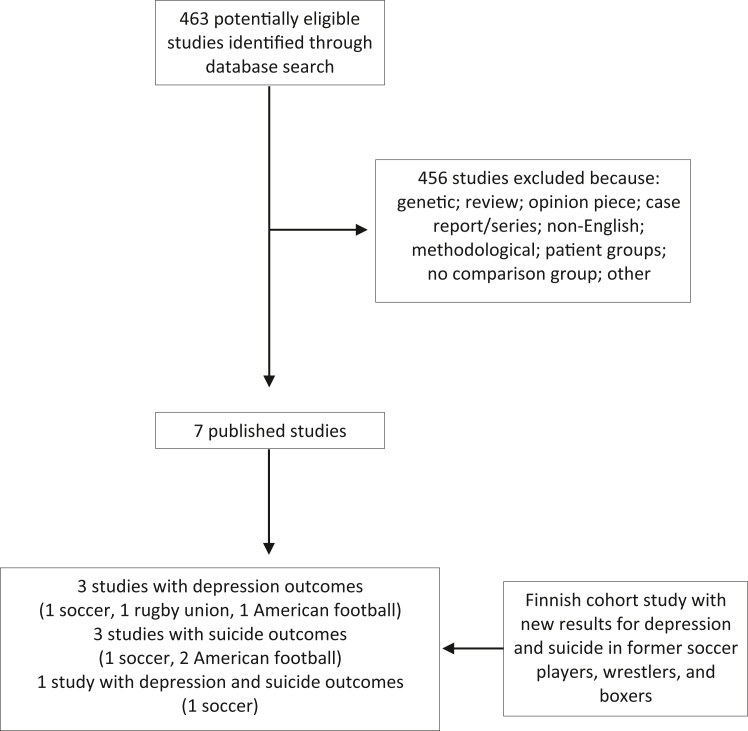
**Study selection: systematic review**.

Our systematic review retrieved 463 potentially eligible published articles of which 7 met the inclusion criteria (Fig. 1).^8^, ^9^, ^10^, ^11^^,^^15^^,^^31^^,^^32^ The characteristics of the included cohort studies are summarised in Table 3. Three featured depression only as the outcome of interest,^11^^,^^15^^,^^32^ 3 reported exclusively on suicide,^9^^,^^10^^,^^31^ and 1 captured both endpoints.^8^ All studies exclusively comprised men and, bar two,^15^^,^^32^ sampled former professional athletes. The number of events was low, ranging from 21^32^ to 38^8^ for depression, and 8^10^ to 19^8^ for suicide. Of the 7 retrieved studies, 3 were evaluated as being of high quality.^8^^,^^9^^,^^31^

**Table 3:** Former participation in contact sports in relation to risk of depression and suicide: characteristics of studies included in the meta-analysis.

Author (year of publication), study design	Exposed (unexposed group)	Country	Years active	Follow-up duration	Number of exposed individuals (number of cases)	Number of unexposed individuals (number of cases)	Risk ratio (95% confidence interval) for exposed versus unexposed
**Soccer**							
Taioli (2007),^10^ retrospective cohort study	Former professional athletes (general population)	Italy	1975–2003	0–28 years	5209 men (8 suicide deaths)	NR (9.92 expected)	Suicide: 0.81 (0.35, 1.59)
Fernandes et al. (2019),^11^ retrospective cohort study	Former professional athletes (orthopaedic patients)	England	NR	NR	572 men (33 depression ‘caseness’)	500 (28 depression ‘caseness’)	Depression: 1.03 (0.61, 1.73)
Russell et al. (2020),^8^ retrospective cohort study	Former professional athletes (general population)	Scotland	NR	Median 18 years	7676 men (38 depression cases; 19 suicide cases)	23,028 males from the general population (169 depression cases; 93 suicide cases)	Depression: 0.64 (0.44, 0.92) Suicide: 0.69 (0.25, 1.87)
**American Football**							
Lehman et al. (2016),^9^ retrospective cohort study	Former professional athletes (general population)	USA	1959–1988	Maximum 34 years	3439 (12 suicide deaths)	NR (25.6 expected)	Suicide: 0.47 (0.24, 0.82)
Lincoln et al. (2018),^31^ retrospective cohort study	Former professional athletes (general population)	USA	1986–2012	2–28 years	9778 (20 suicide deaths)	NR (34.5 expected)	Suicide: 0.58 (0.35, 0.90)
Phelps et al. (2022),^15^ retrospective cohort study	Former high school athletes (general population)	USA	1964–1980	38–55	216 (30 depression ‘caseness’)	638 (94 depression ‘caseness’)	Depression: 0.93 (0.60, 1.45)
**Rugby union**							
Decq et al. (2016),^32^ retrospective cohort study	Former amateur athletes (general population)	France	1985–1990	NR	239 (21 depression ‘caseness’)	138 (8 depression ‘caseness’)	Depression: 1.56 (0.67, 3.64)

NR, not reported.

**Fig. 2:**
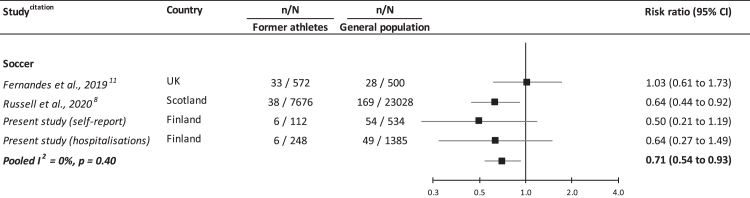
**Risk ratios (95% confidence intervals) for the relation of former participation in soccer with depression: meta-analysis**.

In analyses of specific sports, we aggregated results when there was a minimum of two studies capturing the same activity. We found a lower risk of depression amongst former soccer players relative to control groups (4 studies: 0.71 [0.54, 0.93]; I^2^ = 0%, p-value = 0.40) (Fig. 2). When we stratified according to studies sampling former professionals (2 studies: 0.78 [0.49, 1.24]; I^2^ = 53%, p-value = 0.06)^8^^,^^11^ and amateurs in the Finnish cohort (2 studies: 0.57 [0.31, 1.04]; I^2^ = 0%, p-value = 0.69), there was some suggestion of a lower risk of depression in both groups but not at conventional levels of statistical significance. There was only one study of depression in retired American football players,^15^ and here the prevalence of depression in this cohort of former high school participants was not appreciably different to that of the general population (Table 3).

**Fig. 3:**
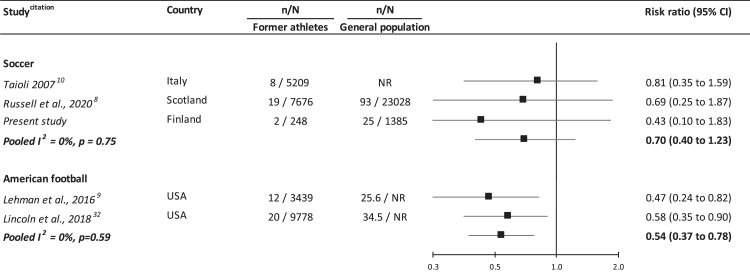
**Risk ratios (95% confidence intervals) for the relation of former participation in contact sports with suicide: meta-analysis**.

In analyses of studies with data on suicide (Fig. 3), retired soccer players had somewhat lower rates than the general population but not significantly so (3 studies: 0.70 [0.40, 1.23]; I^2^ = 0%, p-value = 0.75). There was also no suggestion of a differential effect for former amateurs (0.43 [0.10, 1.83])^8^^,^^11^ versus professional players (2 studies: 0.76 [0.42, 1.40], I^2^ = 0%, p-value = 0.80). In the two studies suicide amongst retired American football players, however, a background in this contact sport was associated with protection against this behaviour (2 studies: 0.54 [0.37, 0.78]; I^2^ = 0%, p-value = 0.59).

## Discussion

In the present report, we aggregated new results from analyses of a cohort study of former contact sports participants with those from the extant literature. With the caveats that the evidence base is modest in scale and confined to men, there was no suggestion that retired soccer and American football players had poorer mental health than the general population. Rather, we found that, at follow-up, erstwhile soccer players appeared to have a lower risk of depression relative to general population controls, while former American football players seemed to experience some protection against suicide.

These epidemiological observations run counter to those made in case series of athletes from contact sports who, at autopsy, were found to be seemingly affected by a combination of chronic traumatic encephalopathy, depression, and suicidal tendencies,^5^^,^^6^ although this interpretation, particularly in the absence of an unexposed comparator group, is readily challenged.^7^ In a separate body of literature, traumatic brain injury requiring hospitalisation has been linked to a greater future occurrence of depression and suicide in large scale cohort studies.^1^^,^^2^ It may be that head impact in the contact sports included herein is of insufficient severity to precipitate long-term depression and suicide. Alternatively, post-retirement level of physical activity in elite athletes is seemingly higher than the general population,^33^ and, as such, the apparent preventative effect of long-standing patterns of physical exertion against depression^34^ and suicidal ideation^35^ may be compensating for the deleterious effect, if any, of low level head trauma.

The present study has its strengths, including being the first synthesis of depression risk in former participants from contact sports, and one that incorporates new cohort study data. It is not, however, without its limitations. First, all included studies exclusively sample men. There is some evidence of sex differentials in other risk factors for depression^36^ and suicide,^37^ so the extent to which the present findings for soccer can be generalised to women is moot. Second, the findings of a meta-analysis are only as strong as the methodological quality of the studies on which it draws and, although half the studies were judged to be of high grade, all data were nonetheless observational. With conventional trials in this field being unviable ethically and perhaps logistically, an advance on current evidence may be the use of natural experiments. These could include the impact on depression or suicide risk pre- and post-introduction of compulsory protective equipment such as change in the composition of the soccer ball from leather to plastic (1986–present) which, despite the same dry weight, would have resulted in a lighter ball in rain-soaked conditions—in European countries, soccer is played in winter—or the introduction of head gear in amateur boxing (1984–2016). Third, none of the included studies had data on actual head impacts; instead, sporting background was used as a proxy. There is empirical evidence, however, of a higher occurrence of head trauma in contact sports groups versus control populations,^38^ and we reason that head trauma itself is likely to be positively correlated with the occurrence of lower-intensity head impacts. Fourth, in an analytical sample comprising individuals who were alive in 1970 when surveillance for depression and suicide began (N = 3391) in the Finnish cohort, there was inevitable loss to follow-up. This was attributable to questionnaire non-response rather than a failure to link study members to health registries. We therefore conducted analyses on a non-missing dataset. Lastly, in some of the retrieved studies, and the Finnish cohort study in particular, there is a gap between the end of the study members’ careers and the start of surveillance for depression and suicide. Inevitably, events will have been omitted, as they would have been for depression. For this to have had an impact on the computation of point estimates, outcome ascertainment would have needed to be differential with respect to our exposure, sports characterised by head impacts, and we are unclear if this is the case.

In conclusion, based on a modest number of studies exclusively comprising men who were largely from professional backgrounds, retired soccer players had a lower risk of depression and former American football players had a lower risk of suicide at follow-up. Whether these findings are generalisable to women requires testing.

## Contributors

GDB generated the idea for the paper; formulated the plan for analyses of the cohort data; conducted the literature search for the systematic review; extracted results, prepared tables and figures; and drafted the manuscript. PF conducted the literature search for the systematic review; carried out the meta-analyses; prepared figures; and edited the manuscript. UMK and SJS initiated the Finnish cohort study; designed data collection; accessed and verified the cohort data; and edited the manuscript. JK designed data collection in the Finnish cohort study; formulated the plan for analyses of cohort data; accessed, verified and analysed the Finnish cohort data; and edited the manuscript.

## Data sharing statement

*Bona fide* interested parties should contact UMK and SJS regarding access to the Finnish cohort study data.

## Declaration of interests

None.

## Appendix A Supplementary data

Supplemental Tables S1–S3 and Supplemental Box S1
